# Recurrent Refractory Non-Dupuytren Contracture in Females After Limited Fasciectomy

**DOI:** 10.7759/cureus.74956

**Published:** 2024-12-02

**Authors:** Yuta Takasu, Koji Moriya, Takuma Kuroda, Hisao Koda, Naoto Tsubokawa

**Affiliations:** 1 Department of Orthopaedic Surgery, Tottori University Hospital, Yonago, JPN; 2 Department of Orthopaedics, Niigata Hand Surgery Foundation, Seiro-machi, JPN

**Keywords:** limited fasciectomy, non-dupuytren contracture, post operative treatment, recurrence, splint

## Abstract

Non-Dupuytren’s contracture, a cord-like structure formed because of trauma or surgery, rarely requires surgery. An 81-year-old woman underwent flexor tendon sheath release for right middle finger snapping and flexor tenolysis for postoperative complications. At the referral, a cord existed between the base of the middle finger and the mid-palm, and a 50° extension deficit of the metacarpophalangeal joint was noted. We performed a third operation. Limited fasciectomy could not achieve full extension; therefore, flexor tenolysis and joint mobilization were additionally performed intraoperatively. Early range of motion exercises were initiated; however, the patient underwent reoperation due to recurrence. Although the cord and bowstringing flexor digitorum superficialis tendon were excised, the flexion contracture remained. Hence, resection of the volar capsule was added intraoperatively. Nevertheless, the flexion contracture recurred. In planning surgery for a patient with non-Dupuytren contracture, considering the possibility of recurrence may be necessary.

## Introduction

Flexion contracture can lead to various functional challenges, including difficulties with washing, picking up objects, wearing gloves, holding items, and placing hands in pockets. These challenges can significantly reduce a patient's quality of life [1]. Dupuytren's contracture is a common cause of flexion contracture, often progressive and requiring surgery. In contrast, flexion contracture caused by a longitudinal fibrous band (cord) following trauma or surgery differs from Dupuytren’s contracture. This non-Dupuytren's contracture is usually mild and non-progressive; therefore, surgery is rarely required [2]. Here, we report a case of recurring flexion contracture of the metacarpophalangeal (MCP) joint following tendon sheath release for middle finger snapping and suggest a postoperative management approach.

## Case presentation

**Figure 1:**
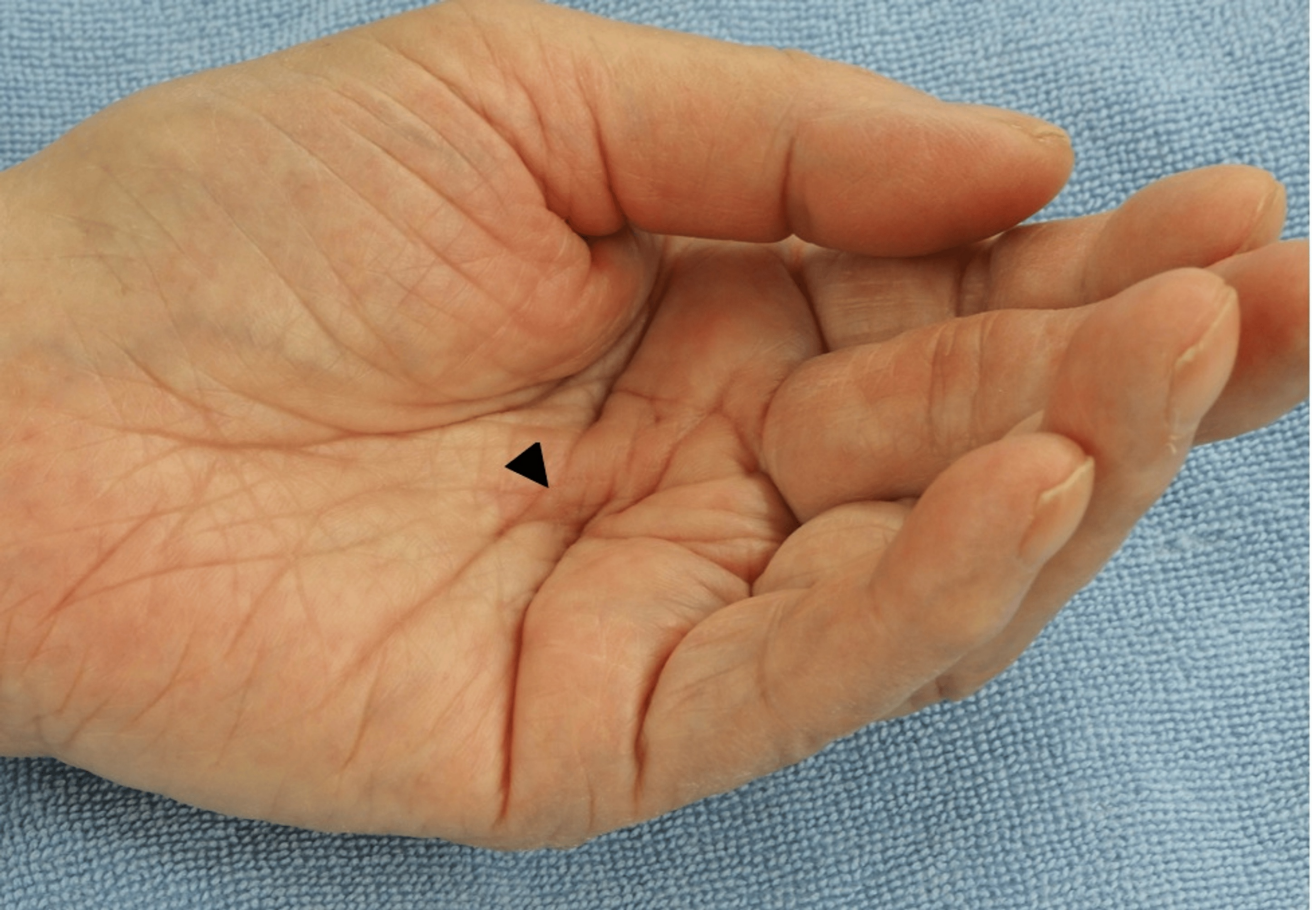
Preoperative findings. A cord (arrowhead) exists between the proximal part of the middle finger and the mid-palm.

**Figure 2:**
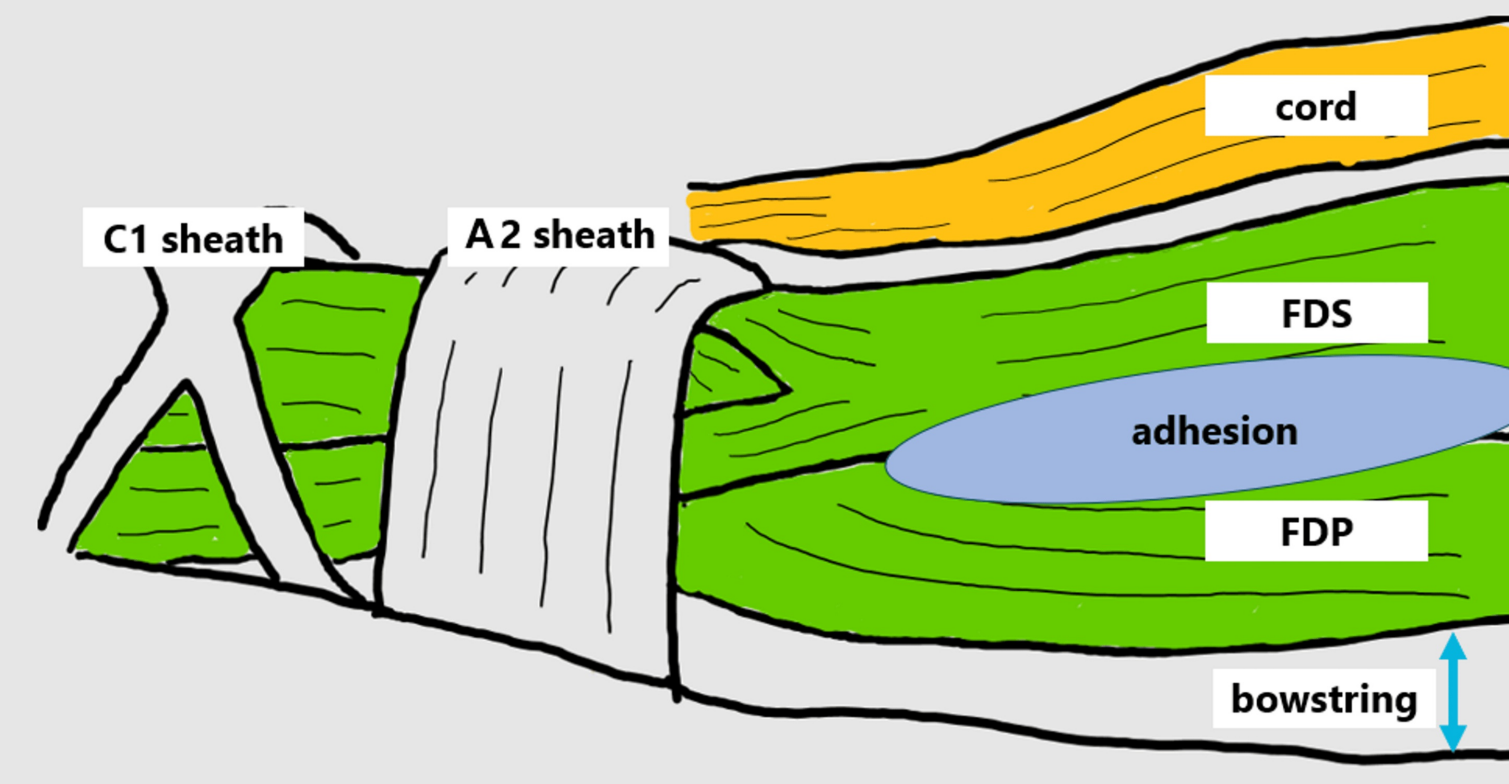
Intraoperative findings. Lateral view during the third operation. The A1 sheath had been excised during previous tenolysis. Both the FDS and FDP tendons were adhered and bowstringed after cord resection. Image Credits: Yuta Takasu, Author. FDS: Flexor digitorum superficialis; FDP: Flexor digitorum profundus.

**Figure 3:**
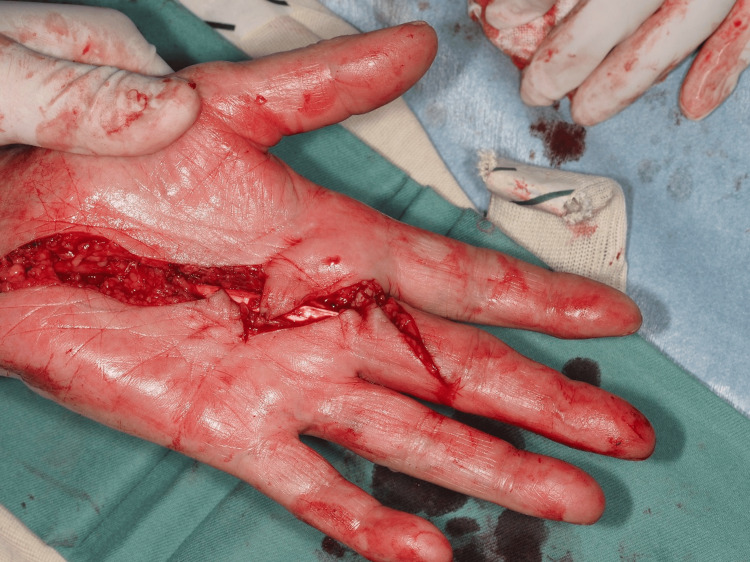
After cord resection and joint mobilization. Full extension of the MCP joint was achieved by flexor tenolysis and transverse incision of the volar plate at the MCP joint of the middle finger. MCP: Metacarpophalangeal.

An 81-year-old woman with diabetes mellitus underwent flexor tendon sheath release for left middle finger snapping at another hospital. Nine months post-surgery, flexor tenolysis was performed for flexion contracture of the MCP joint. Twenty months after the initial procedure, the patient presented at our hospital with a recurrence of flexion contracture. A cord extended from the MCP joint of the right middle finger to the mid-palm, resulting in a 50° extension deficit at the MCP joint (Figure 1). Active flexion was limited to 50° at the proximal interphalangeal (PIP) joint and 40° at the distal interphalangeal (DIP) joint. The patient was experiencing difficulty in daily life due to the limited flexion and extension of the middle finger. A third surgery was performed to release the flexion contracture. We exposed the middle finger PIP joint to the distal forearm and excised the cord. Both the flexor digitorum superficialis (FDS) and flexor digitorum profundus (FDP) tendons, which were adhered from the distal flexor retinaculum to the proximal A2 tendon sheath, displayed bowstringing following the cord excision (Figure 2). As full MCP joint extension was unachievable by cord excision alone, flexor tenolysis was performed after a carpal tunnel release; however, the flexion contracture persisted. We incised the MCP joint's volar plate transversely, achieving full extension (Figure 3)

**Figure 4:**
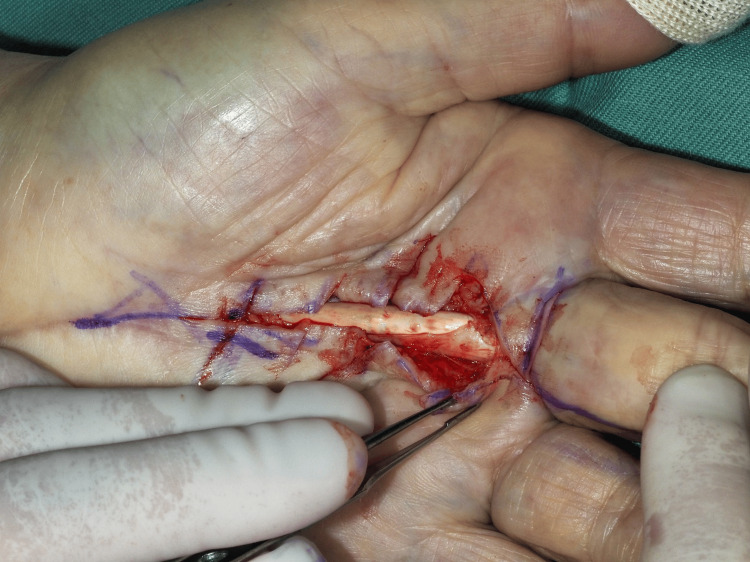
Intraoperative findings in the fourth operation. Bowstringing of the flexor digitorum superficialis (FDS) tendon was observed after cord resection.

**Figure 5:**
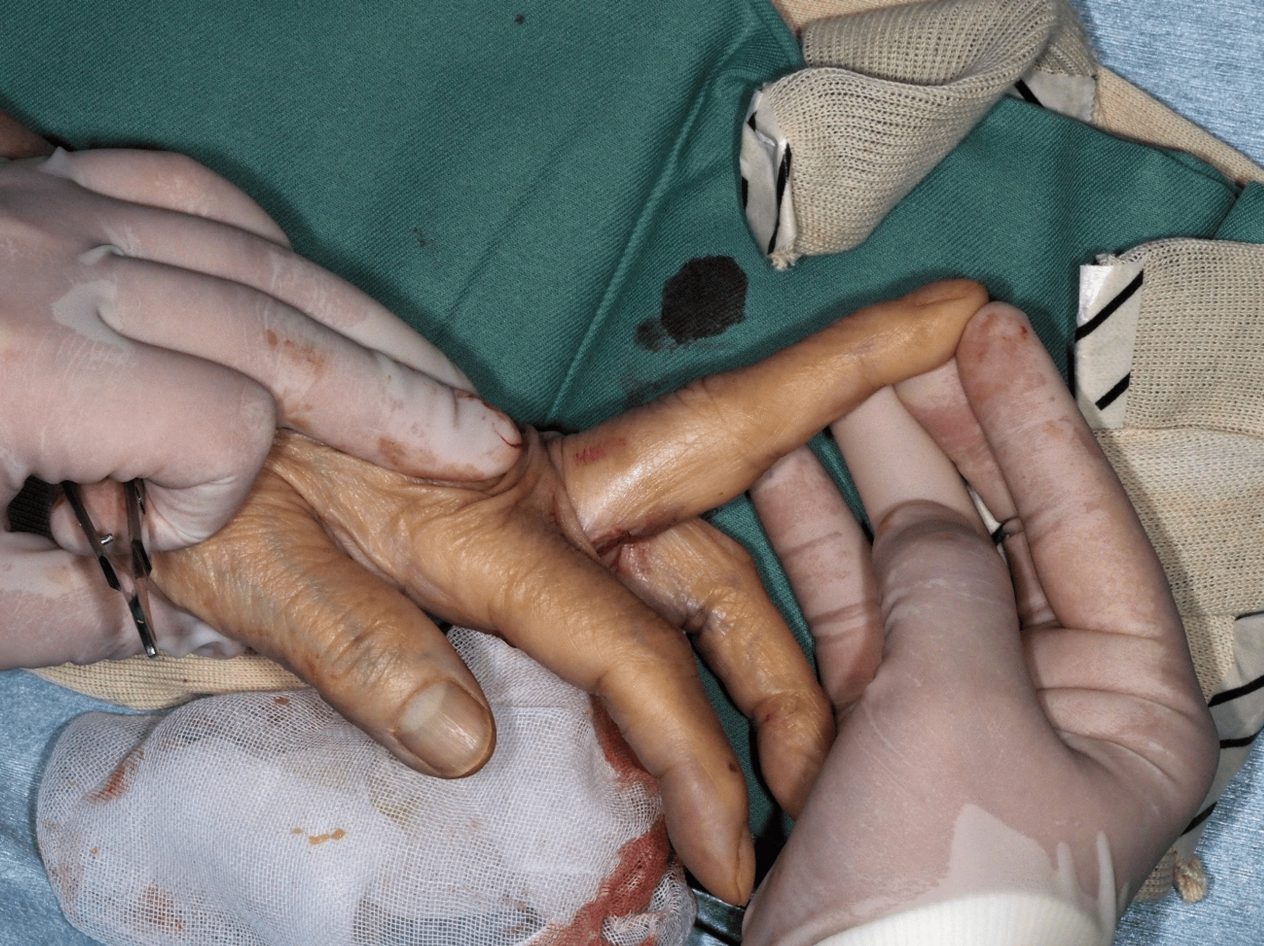
After resection of the cord and FDS tendon, and mobilization. Full extension of the metacarpophalangeal (MCP) joint was achieved after resection of the cord, FDS tendon, and the volar capsule at the MCP joint of the middle finger. FDS: Flexor digitorum superficialis.

Despite early postoperative range of motion (ROM) exercises, flexion contracture recurred within six months, and the patient requested a fourth surgery. The cord was exposed through a zigzag incision with a triangular flap on the radial side of the base of the middle finger. The palmar fascia appeared band-like and thickened, with the FDS tendon bowstringing beneath the cord (Figure 4). Although both the cord and FDS tendon were excised, MCP joint flexion contracture remained. The volar plate and capsule were resected to mobilize the MCP joint (Figure 5). Z-plasty with a triangular flap was performed to close the wound and reduce palmar skin tension. Four months postoperatively, flexion contracture recurred, gradually evolving into a swan-neck deformity. The patient declined further surgical intervention and continues with ROM exercises.

## Discussion

This case developed flexion contracture resulting from a longitudinal fibrous band following minor surgery. Despite interventions such as limited fasciectomy, tenolysis, joint mobilization, and intensive hand therapy, flexion contracture of the affected finger recurred. Initially, this case was believed to be non-Dupuytren’s contracture.

Elliot D and Ragoowansi R characterized trauma- or surgery-induced Dupuytren's contracture (Table 1) [3]. This non-Dupuytren’s contracture, or post-traumatic disease of the palmar fascia (PTDPF), is differentiated from classic Dupuytren’s contracture. Common PTDPF causes include trauma, such as palmar lacerations or fractures, and surgeries like carpal tunnel or tendon sheath release, as seen in our patient. Unlike Dupuytren’s contracture, PTDPF generally shows limited cord progression, with minimal PIP joint contracture [2]. Most PTDPF cases resolve without surgery, with only 20% requiring fasciectomy (Table 2) [2].

**Table 1:** Criteria for the recognition of Dupuytren’s contracture after acute injury. Elliot D and Ragoowansi R’s criteria for the recognition of Dupuytren’s contracture after acute injury [3].

S. No.	Elliot D and Ragoowansi R’s criteria
1	There is objective evidence of injury with no evidence of Dupuytren's disease prior to the injury.
2	The injury was within the same hand, wrist or forearm as the first hand to develop disease
3	The patient may be of any age and may or may not exhibit conditions predisposing to Dupuytren's disease or indicative of a diathesis.
4	Disease appears within one year of injury.
5	A single nodule or band appears first in the palm of the injured hand.
6	Disease commonly remains limited to the part of the hand initially involved but may progress within the same hand or to the other hand and may occasionally become significant in degree.

**Table 2:** Percentage of surgeries performed for non-Dupuytren's contracture and recurrence rates.

Authors	Number of cases	Requiring surgery (%)	Recurrence (%)
Elliot D and Ragoowansi R [3]	52	6 (12)	4 (8)
Rayan GM and Moore J [4]	39	7 (18)	0
Abe Y et al. [5]	16	5 (31)	Not reported

The pathogenesis of non-Dupuytren’s contracture involves a series of events where minor palmar trauma induces inflammation, leading to excessive fibroblast-driven collagen production mediated by cytokines like interleukin-1. Mechanical stress during rehabilitation further exacerbates inflammation [2]. Unlike Dupuytren’s contracture, the non-Dupuytren's cord contains no myofibroblasts [4]. Collagen synthesis peaks around six months post-injury during wound healing [6].

Given the histopathological features of non-Dupuytren's contracture, we propose that managing local inflammation and inhibiting excessive collagen production may be more effective than surgical treatment alone in preventing recurrence. Nonsteroidal anti-inflammatory drugs, steroid ointments, and tranilast may help control local inflammation and collagen synthesis through fibroblast inhibition [7]. Moreover, hand therapy plays a critical role in achieving optimal ROM without triggering an inflammatory response. Based on our experience, it is essential to incorporate rest during intense inflammation, avoid excessive extension that may provoke inflammation, and use cooling post-exercise. Additionally, an extension night splint from the early postoperative period up to six months may help prevent flexion contracture progression during scar formation.

## Conclusions

This case report suggests that surgical treatment for non-Dupuytren's contracture may result in multiple additional surgeries for recurrent flexion contracture in certain cases. When planning surgery for non-Dupuytren's contracture, it is crucial to consider the risk of recurrence. Regardless of whether it is the initial onset or a recurrence, hand therapy that avoids provoking inflammation and splinting to prevent the progression of flexion contracture are essential for managing non-Dupuytren’s contracture.
